# A survey of the adaptive immune genes of the polka-dot batfish *Ogcocephalus cubifrons*

**DOI:** 10.1186/s12865-023-00557-0

**Published:** 2023-07-21

**Authors:** Jeremy B. Swann, Christiane Grammer, Michael Schorpp, Thomas Boehm

**Affiliations:** 1 Department of Developmental Immunology, Max Planck-Institute of Immunobiology and Epigenetics, Stübeweg 51, D-79108 Freiburg, Germany; 2 Faculty of Medicine, University of Freiburg, Freiburg, Germany

**Keywords:** Anglerfish, Batfish, Adaptive immunity, Immune evolution

## Abstract

**Background:**

The anglerfish, belonging to the teleost order *Lophiiformes*, are a diverse and species-rich group of fish that are known to exhibit a number of unique morphological, reproductive and immunological adaptations. Work to date has identified the loss of specific adaptive immune components in two of the five *Lophiiformes* sub-orders (*Lophioidei* and *Ceratioidei*), while no anomalies have been identified to date in two other sub-orders, *Antennaroidei* and *Chaunacoidei*. The immunogenome of the fifth sub-order, *Ogcocephaloidei* has not yet been investigated, and we have therefore used whole genome shotgun sequencing, combined with RNA-seq, to survey the adaptive immune capabilities of the polka-dot batfish, *O. cubifrons*, as a representative of this as yet unexplored sub-order.

**Results:**

We find that the *O. cubifrons* genome encodes the core genes needed to mount adaptive T and B cell responses. These genes include those necessary for rearranging and editing antigen receptors, the antigen receptors themselves; as well as the co-receptors, signalling molecules, and antigen presenting molecules (both class I and class II) needed for B cell and T cell development and activation.

**Conclusions:**

From an immune perspective, the polka-dot batfish has a canonical complement of adaptive immune genes, and does not exhibit any of the adaptive immune changes previously identified in monkfish and oceanic anglerfish.

**Supplementary Information:**

The online version contains supplementary material available at 10.1186/s12865-023-00557-0.

## Background

The anglerfish, so named due to their use of an esca (lure) to attract prey, are a large grouping of fish belonging to the teleost order *Lophiiformes*, a morphologically diverse and species-rich order that exhibits many fascinating biological characteristics. One of these characteristics is the make-up of the adaptive immune system, which for several anglerfish species has been shown to differ significantly from the canonical form present in most other jawed vertebrates.

The *Lophiiformes* are currently divided into five suborders: *Lophioidei* (monkfish and goosefish), *Antennaroidei* (frogfish), *Ogcocephaloidei* (batfish), *Chaunacoidei* (sea toads) and *Ceratiodei* (deep-sea anglers) [1, 2]. Remarkably, genome analysis has revealed that species within the sub-orders *Lophioidei* [3] and *Ceratiodei* [4] exhibit substantial modifications to their adaptive immune capabilities. In the case of the *Lophioidei*, analysis of *Lophius piscatorius* has revealed that this species lacks the MHC2 antigen presentation pathway; the MHC2 alpha and beta genes themselves, as well as the genes encoding CD74 (required for peptide loading and trafficking of MHC2 complexes), and the MHC2 co-receptor CD4 have all been lost [3] (see Supplementary Fig. 1 for an overview of adaptive immune gene losses).

Deep-sea anglerfish utilise a peculiar method of reproduction referred to as sexual parasitism, in which pair formation for reproduction is mediated by physical attachments, with a male anglerfish attaching to a female partner using his mouth, a trait which is thought to be common to all members of the ceratioid suborder. Within the ceratioids there is a range of pairing behaviours, from temporary attachment (where transient interactions between males and females occur during breeding), permanent exclusive attachment (species in which a female permanently pairs with a single male via tissue fusion), to permanent multi-attachment (species in which a female permanently pairs with multiple males via tissue fusion) [2]. This continuum correlates with the loss of adaptive immune genes, ranging from relatively modest changes, such as the loss of *aicda* (activation-induced cytidine deaminase) in species that form temporary attachments, through to the most extreme change, the loss of RAG-mediated antigen receptor assembly in some species that form permanent multiple attachments [4].

The loss of immune genes in the *Lophioidei* and *Ceratioidei* sub-orders poses a conundrum: while within the ceratioid sub-order the loss of immune genes correlates with mating behaviour, the lophioids do not form physical pair bonds, so in this instance the loss of the MHC2-pathway cannot be readily attributed to reproductive mode. This observation may indicate that the drivers leading to loss of adaptive immune system components differ in these two instances, and raises the question of how widespread the loss of adaptive immune genes is within the *Lophiiformes* order. To date the immunogenomes of representative members of the *Lophioidei*, *Antennaroidei*, *Chaunacoidei* and *Ceratioidei* have been examined, however to our knowledge the adaptive immune facilities of the *Ogcocephaloidei* have not yet been explored. An overview of the immune genes present in this sub-order is therefore desirable to understand the scope and extent of the adaptive immune changes occurring within the *Lophiiformes*.

Over time many approaches have been used to determine the evolutionary relationships among the *Lophiiformes* sub-orders, including morphological/anatomical characteristics [1, 5, 6], mitochondrial genes [7], mitochondrial genomes [8], combinations of both nuclear and mitochondrial genes [9, 10], purely nuclear genes [11], molecular characteristics [12], and most recently ultraconserved genomic elements [13]. These approaches have yielded a variety of potential subordinal relationships (reviewed in [13]), however almost all of the proposed phylogenies indicate that the *Lophioidei* and *Ceratioidei*, the two sub-orders with known losses of adaptive immune genes, are relatively distantly related. The position of the *Ogcocepahloidei* within the *Lophiiformes* is somewhat unsettled, as while molecular phylogenies indicate that this sub-order is most closely related to the *Antennarioidei* [9–11, 13], phylogenies based on morphological characteristics propose a close relationship to the *Ceratioidei* [5, 6]. Given this uncertainty we resolved to investigate the immunogenome of a representative of the *Ogcocephaloidei*, to determine if members of this sub-order exhibit any overt modifications to their adaptive immune gene complement. Reports from experienced aquarists have indicated that batfish commonly suffer from high parasitic loads, and that aggressive use of anti-parasitic medication is often required in order to successfully adapt wild-caught individuals to captivity [14], further prompting interest in understanding the immunocompetence of this suborder.

We report here the survey of the adaptive immune genes present in the polka-dot batfish, *Ocgocephalus cubifrons*, and conclude that this species retains the core adaptive immune genes expected in a canonical jawed-vertebrate immune system, including the essential genes for mounting MHC1- and MHC2-mediated T cell responses, as well as B cell-mediated antibody responses.

## Results

### Essential genes enabling somatic modification of antigen receptors are present

**Fig. 1:**
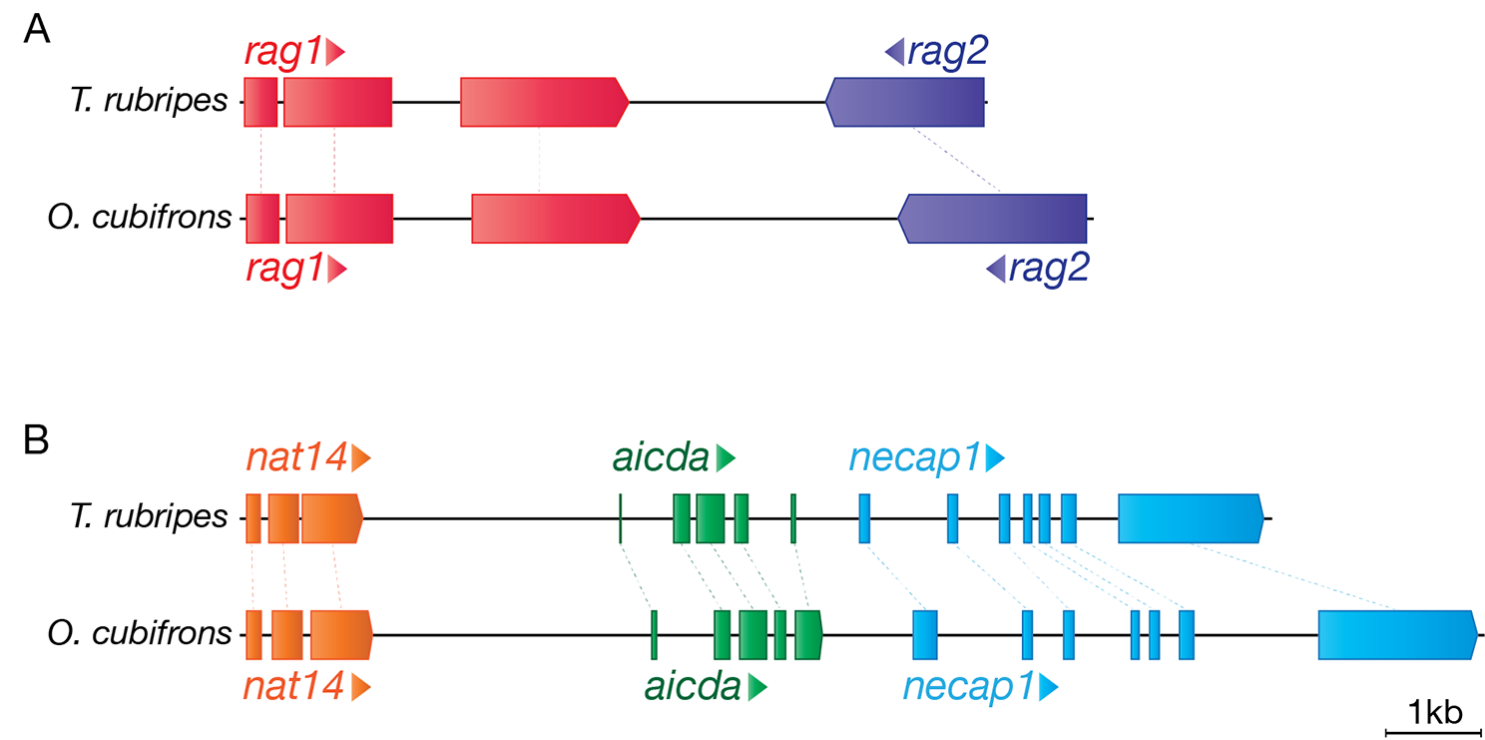
Genomic configuration of the rag and aicda loci. The configuration of the *rag1*-*rag2* (**A**) and the *aicda* (**B**) loci from *O. cubifrons* is depicted, relative to the corresponding loci in *T. rubripes.* In *O. cubifrons* the *rag1* and *rag2* genes are found in a tail-to-tail configuration, with the *rag1* gene comprised of three coding exons (shown in red), while the *rag2* gene has a single coding exon (dark blue). The *O. cubifrons aicda* gene is comprised of five coding exons (**B**, green), and is flanked by *nat14* (orange) and *necap1* (light blue) genes, as is the case in *T. rubripes*. The depicted scale bar applies to both (**A**) and (**B**). Only coding exons are indicated. Sequences for the *O. cubifrons rag*- and *aicda*- loci are available under the GenBank accession numbers OP856783 and OP856782 respectively

The generation of T cell and B cell repertoires is dependent on the *rag1* and *rag2* genes, which are essential for the somatic rearrangement of B and T cell receptors via VDJ recombination. These genes have been lost in some ceratioids, and we therefore began our survey by testing for the presence of rag genes in the genome of *O. cubifrons*. Genomic DNA sequencing revealed the presence of both *rag1* and *rag2* genes in the typical tail to tail orientation (Fig. 1A), and expression of both genes could be detected by RNA-seq on thymus tissue (Table 1). In addition, we also detected expression of a terminal deoxynucleotidyltransferase (*dntt*) orthologue in *O. cubifrons* thymus (Table 1). Phylogenetic analysis (Supplementary Fig. 2) substantiated the identity of the proposed dntt orthologue, distinguishing it from the closely related polymerase µ [15]; the expression of *dntt* transcripts in the thymus of *O. cubifrons* therefore indicates that insertion of non-templated nucleotides likely contributes to junctional diversity during RAG-mediated VDJ-recombination in this species.

**Table 1:** O. cubifrons orthologues identified in this study. Adaptive immunity-related genes are listed, together with the GenBank accession number for the *O. cubifrons* cDNA sequences assembled from RNA-seq data

Gene	*O. cubifrons* cDNA accession #	T. rubripes orthologue as top hit? (E value)^a^
*aicda*	OP856755	yes (0.0)
*b2m1*	OP856756	yes (5e-42)
*b2m2*	OP856757	yes (6e-37)
*cd3e*	OP856758	yes (6e-38)
*cd3gd*	OP856759	yes (2e-35)
*cd3z*	OP856760	yes (4e-64)
*cd4.1* ^*b*^	OP856761	yes (3e-115)
*cd4.2*	OP856762	yes (9e-78)
*cd8a*	OP856763	yes (7e-46)
*cd8b*	OP856764	yes (4e-58)
*cd74*	OP856765	yes (4e-37)
*cd79a*	OP856766	yes (2e-63)
*cd79b*	OP856767	yes (2e-56)
*dntt*	OP856768	yes (0.0)
*ighd* ^*c*^	OP856769	yes (0.0)
*Ighm* ^*c*^	OP856770	yes (2e-172)
*lck*	OP856771	yes (0.0)
*mhc1* ^*d*^	OP856772	yes (5e-129)
*mhc2a*	OP856773	yes (1e-88)
*mhc2b* ^*e*^	OP856774	yes (e3-103)
*polm*	OQ476078	yes (0.0)
*rag1*	OP856775	yes (0.0)
*rag2*	OP856776	yes (0.0)
*syk*	OP856777	yes (0.0)
*tap1*	OP856778	yes (0.0)
*tap2*	OP856779	yes (0.0)
*trac* ^*c*^	OP856780	yes (4e-32)
*trbc* ^*c*^	OP856781	yes (1e-68)

^**a**^ The *O. cubifrons* protein encoded by each cDNA was BLASTed against the *T. rubripes* database, in all cases the top hit was to the expected orthologue; the E value for the top hit is indicated in parentheses

^**b**^Reference sequence uses the “Variant 2” exon 1–3 region discussed in the text

^**C**^The given sequence covers only the constant region of the relevant receptor gene

^**d**^Reference sequence corresponds to clone 1 from Supplementary Fig. 4

^**e**^Reference sequence corresponds to clone 2 from Supplementary Fig. 5

Activation-induced cytidine deaminase (AID, encoded by the *aicda* gene), is required for the process of somatic hypermutation during affinity maturation of antibody responses, and is absent in all of the Ceratioid genomes examined to date. In *O. cubifrons* however, we were able to identify an intact *aicda* gene, with local syntenic conservation of flanking genes (Fig. 1B). Low-level *aicda* expression was also detected in RNA-seq libraries (Table 1). Some fish species, such as the Atlantic cod, have intact *aicda* genes which nevertheless encode AID proteins with little to no catalytic activity [16]. As our sequencing data lacked sufficient coverage to conclusively demonstrate SHM in immunoglobulin sequences, we instead assessed catalytic potential by aligning key regions of the *O. cubifrons* AID protein with a representative set of high (human, zebrafish and pufferfish) and low (atlantic cod) activity AID orthologs. We found that *O. cubifrons* AID exhibits active residues at two key functional sites (Supplementary Fig. 3) indicating that this protein is likely catalytically active.

These observations demonstrate that the *O. cubifrons* genome encodes the core set of somatic DNA modifying genes necessary to generate adaptive T and B cell repertoires.

### Core BCR signalling components are present

**Fig. 2:**
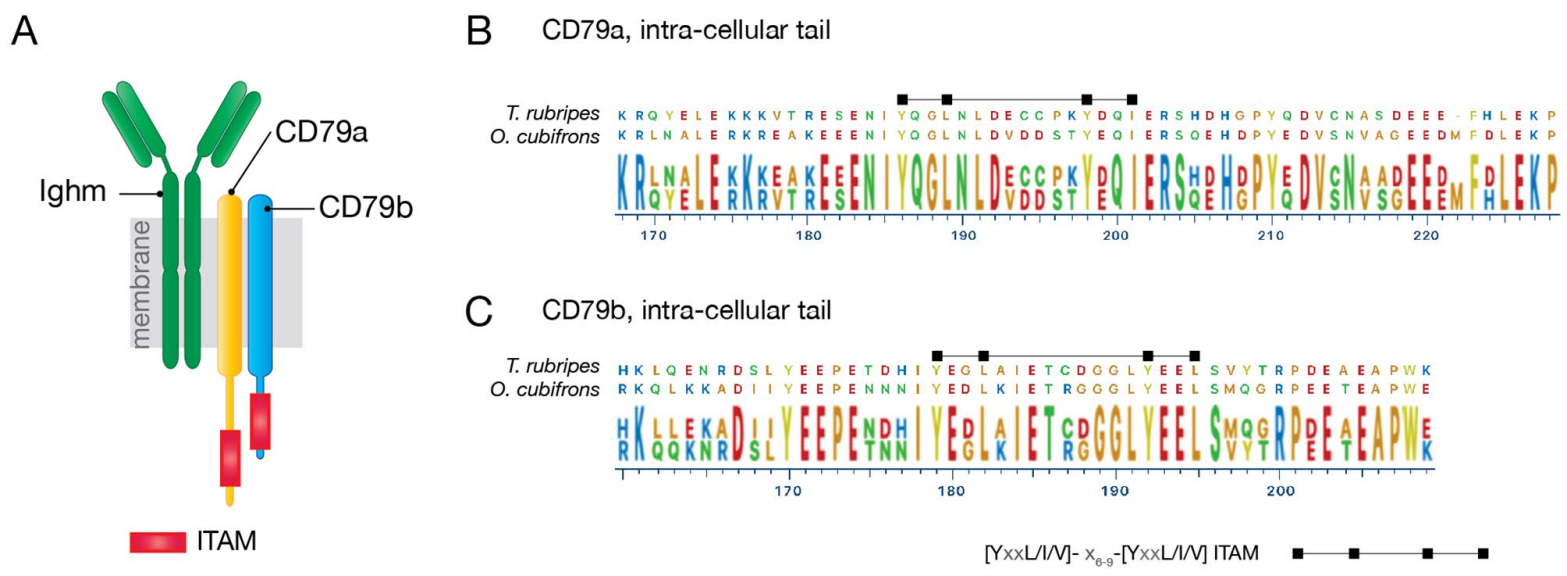
BCR signalling complex components. A schematic of the B cell receptor signalling complex is shown in (**A**). The intra-cellular tail of CD79a and CD79b signalling molecules are shown in (**B**) and (**C**), aligned to the orthologous sequences from *T. rubripes*. The location of ITAMs within the intra-cellular tails is indicated, the ruler below the alignments indicates the amino-acid position within the aligned full-length proteins

Antigen recognition via the B cell receptor (BCR) is an essential step in the generation of an adaptive, antibody-based humoral response. The core BCR signalling complex is composed of the BCR itself, and two accessory signalling components CD79a and CD79b, and we therefore determined the status of these genes in *O. cubifrons*. Screening of the *O. cubifrons* transcriptome revealed expression of both *ighm* and *ighd* immunoglobulin heavy chain genes, as well as *cd79a* and *cd79b* transcripts (Fig. 2A; Table 1). The signalling capacity of the BCR complex is dependent on the presence of immuno-receptor tyrosine-associated activation motifs (ITAMs) present in the intracellular tails of both CD79a and CD79b, and examination of the inferred protein sequence of *O. cubifrons* CD79a and CD79b revealed consensus ITAMs present in the C-terminus of both molecules (Fig. 2B C respectively). The expression of *syk*, a gene encoding a kinase which phosphorylates and interacts with the ITAMs present in CD79a and CD79b during BCR-signalling [17], could also be detected in *O. cubifrons* (Table 1). These results indicate that the core BCR signalling genes are present and expressed in *O. cubifrons*, and together with the presence of the *rag* and *aicda* genes, suggest that these fish have the requisite components to mount adaptive, antibody-mediated immune responses.

### Core TCR signalling complex genes

**Fig. 3:**
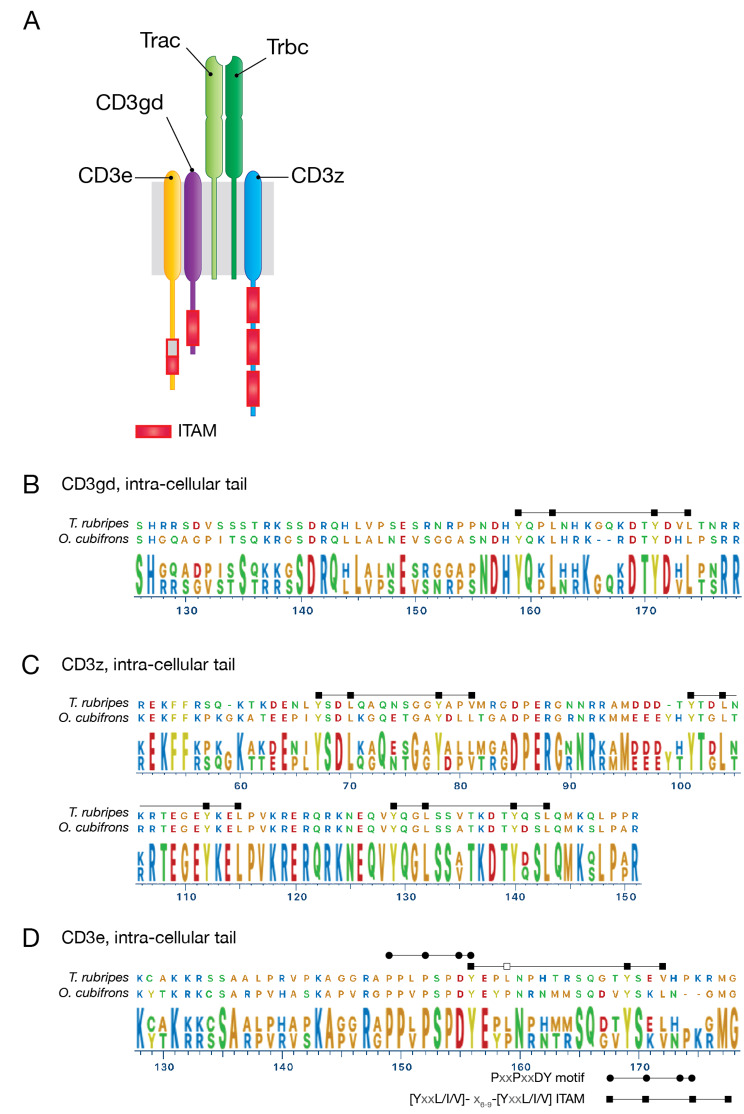
TCR signalling complex components. A schematic of the T cell receptor signalling complex is shown in (**A**). The intra-cellular tail of the CD3gd, CD3z and CD3e signalling chains are shown in (**B**), (**C**) and (**D**) respectively, and are aligned to the orthologous sequences from *T. rubripes*. The location of ITAMs within the intra-cellular tails is indicated, the ruler below the alignments indicates the amino-acid position within the aligned full-length proteins. The presence of a non-consensus amino acid in the first half of the CD3e ITAM is indicated by an open square in the motif marker

The TCR signalling complex is essential for T cell development and antigen-specific cellular responses, and is composed of both antigen recognition components, the heterodimeric a and b TCR chains, and signalling components in the form of CD3 molecules. Expression of both *trac* and *trbc* genes could be readily detected in the *O. cubifrons* thymus (Fig. 3A; Table 1), together with transcripts for *cd3e*, *cd3gd* and *cd3z*. We then examined the intracellular tail of the predicted CD3 proteins for the presence of the ITAMs that are central to TCR-driven signalling, and found that CD3gd and CD3z had the expected number of ITAMs (one and three respectively, Fig. 3B C), all of which conformed to the consensus motif. The putative ITAM in the CD3e sequence however was only a partial match, with a proline, rather than the expected isoleucine, leucine or valine, in the first half of the motif (YxxP, instead of YxxI/L/V; see Fig. 3D). At present, the functional consequence of this modification is unknown, however a proline at position 4 of the CD3e ITAM has been noted in several other anglerfish species [4]. Notably, a PxxPxxDY motif responsible for interactions with Nck [18], and having an important role in TCR-signalling [19] was conserved in the *O. cubifrons* CD3e intracellular tail (Fig. 3D), suggesting that this chain retains at least some signalling capacity. Expression of *lck*, a gene encoding the central kinase involved in ITAM-phosphorylation during TCR-signalling [20], was also detected (Table 1). Overall, these results indicate the presence of a functional TCR-signalling complex in *O. cubifrons*, however variation in the CD3e intracellular tail may alter the overally activity of the complex.

### Class I MHC pathway

**Fig. 4:**
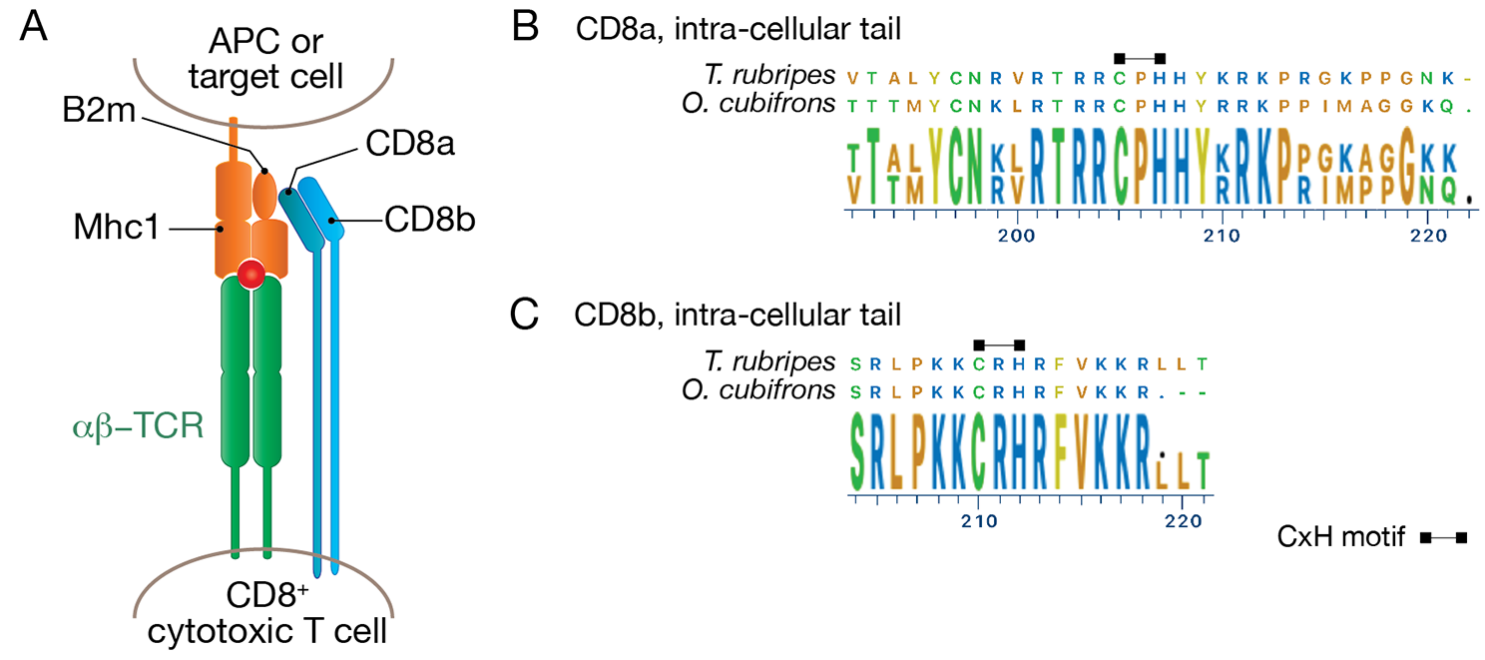
MHC1 pathway components. A schematic of a TCR-MHC1-CD8 complex is shown in (**A**). The intra-cellular tails of the CD8a and CD8b co-receptor chains are shown in (**B**) and (**C**), aligned to the orthologous sequences from *T. rubripes*. The locations of conserved CxH motifs, thought to be necessary for interaction with Lck, are indicated

The MHC1 antigen presentation pathway presents endogenous peptides to T cells via a heterodimer comprised of an MHC heavy chain bound with the accessory protein b2-microglobulin (b2M)[21, 22] (Fig. 4A). BLAST searches of the *O. cubifrons* thymus transcriptome indicated expression of polymorphic *mhc1* genes, and PCR primers were designed to conserved regions of the putative *mhc1* transcripts to assess sequence diversity. Complete MHC1 heavy chain encoding sequences were amplified from thymus tissue of a single individual, cloned into plasmids, and 10 clones were selected at random for Sanger sequencing. Analysis of these 10 clones revealed 8 distinct sequences; however, it has been noted that MHC haplotyping via PCR can artificially inflate allelic diversity [23], and a more stringent assessment based on distinct polymorphic positions is congruent with the presence of at least 3 distinct alleles in the sequenced individual (Supplementary Fig. 4). In addition to expression of polymorphic *mhc1* heavy chain transcripts, interrogation of the *O. cubifrons* transcriptome revealed the expression of two distinct *beta-2-microglobulin* (*b2m)* genes (Table 1), in keeping with detection of multiple *b2m* genes in many fish species [24–26]. Peptide transporters play a critical role in the MHC1-antigen presentation pathway [27–29], and expression of *tap1* and *tap2* genes could also be detected in thymus tissue by RNA-seq (Table 1). Together, these results demonstrate that *O. cubifrons* expresses the core set of genes required for generating peptide-loaded, polymorphic MHC1 complexes.

T cell recognition of peptide/MHC1-complexes is enhanced via the binding of the CD8 co-receptor, which functions as either a CD8ab heterodimer, or as a CD8aa homodimer. Expression of *O. cubifrons cd8a* and *cd8b* orthologues were readily detected in the *O. cubifrons* transcriptome (Table 1). The co-receptor function of CD8 is dependent on the ability to interact with Lck, which in teleosts is thought to occur via a CxH motif present in the co-receptor, and a CxxC motif present in the kinase [30]. Examination of the inferred protein sequences of the *O. cubifrons cd8a* and *cd8b* cDNAs revealed the presence of a CxH motif within the intracellular tail of both molecules (Fig. 4B C), indicating that they have the potential to mediate signalling via Lck binding.

Collectively these results show that the basic molecules required for the presentation of MHC1/peptide-complexes to TCR/CD8-co-receptor expressing T cells are present in *O. cubifrons*.

### Class II MHC pathway

**Fig. 5:**
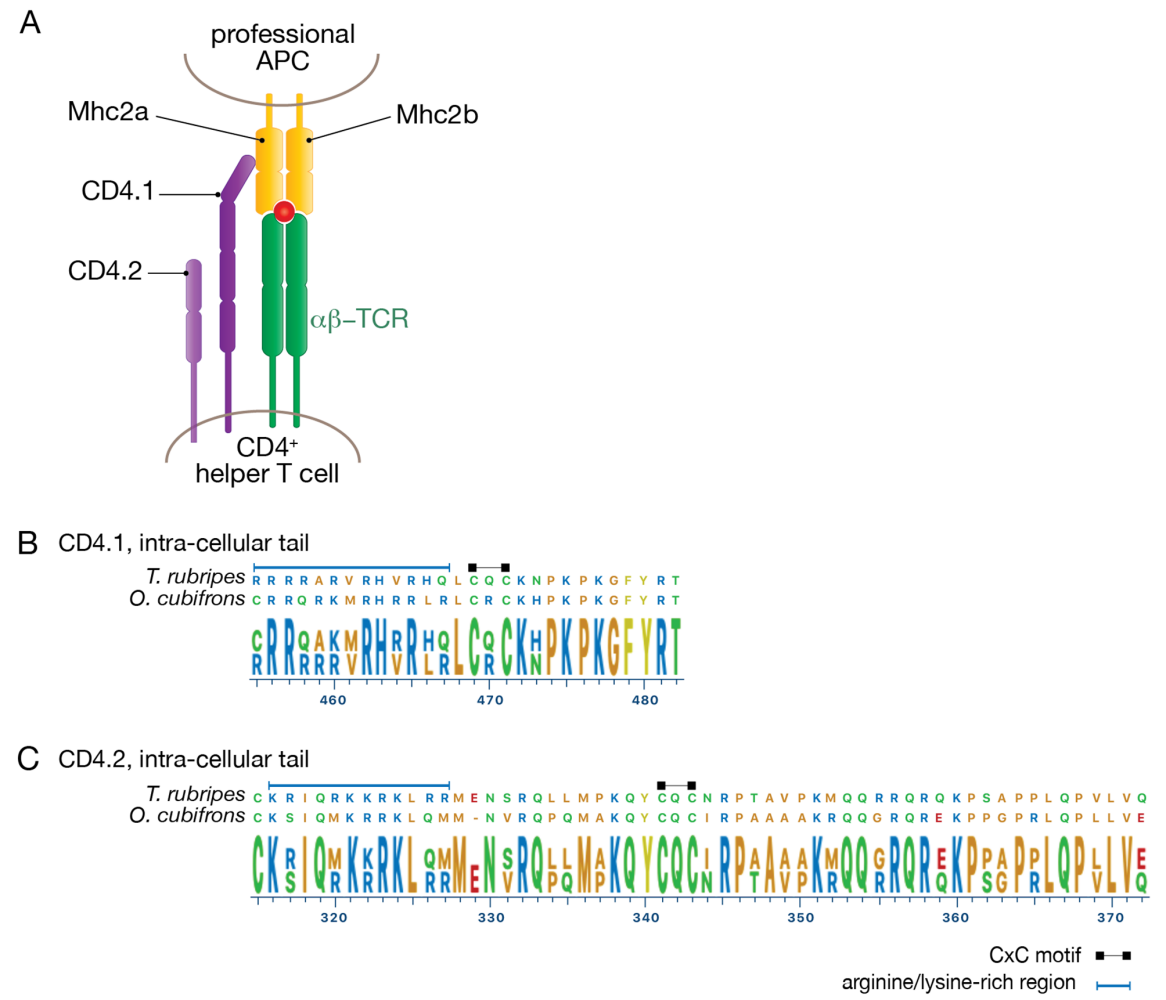
MHC2 pathway components. A schematic of a TCR-MHC2-CD4 complex is shown in (**A**). The intra-cellular tails of the CD4.1 and CD4.2 co-receptor chains are shown in (**B**) and (**C**), aligned to the orthologous sequences from *T. rubripes*. The locations of conserved CxC Lck-interaction motifs (black squares) and arginine/lysine-rich regions (blue line) within the intra-cellular tails are indicated

The MHC2 antigen presentation pathway presents exogenous peptides to T cells via a heterodimer comprised of MHC2 a- and b-chains ([31] and Fig. 5A); components of this pathway have been lost in several species within the Lophiiformes order. We therefore performed BLAST searches to identify expression of representative *mhc2a* and *mhc2b* genes in *O. cubifrons*, and detected expression of both *mhc2a* and *mhc2b* orthologues in thymus transcriptome data (Table 1). Analysis of *mhc2a* transcripts revealed only minimal sequence diversity, however RNA-seq data indicated the presence of polymorphic *mhc2b* alleles. To characterise this diversity, we designed primers binding in conserved regions to amplify the entire coding sequence of an *mhc2b* target from a single individual, and cloned and sequenced the resulting amplicons. In this instance, analysis of 10 randomly selected clones demonstrated the presence of at least two distinct *mhc2b* alleles (Supplementary Fig. 5). Therefore, unlike the situation described in *Lophius* species, *mhc2* genes are retained in *O. cubifrons*. Expression of *cd74*, another MHC2-pathway gene [32, 33] that is absent in *Lophioidei*, could also be readily detected in *O. cubifrons* RNA-seq data (Table 1).

**Fig. 6:**
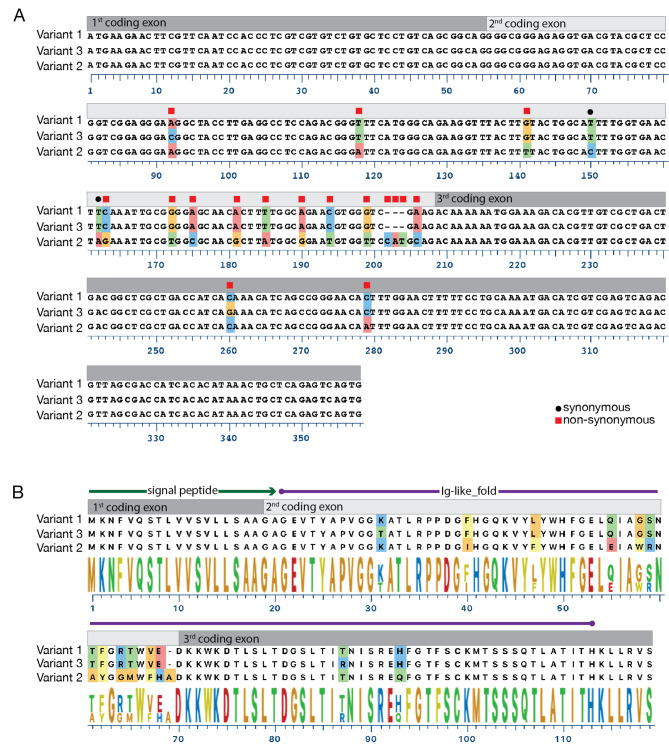
CD4.1 sequence polymorphism in *O. cubifrons*. The sequence of the first three coding exons of three variant *O. cubifrons cd4.1* alleles are shown in (**A**). Bars above the alignment indicate exon position, and shaded nucleotides indicate polymorphic sites. Black circles above the polymorphic positions indicate synonymous sites, red squares indicate non-synonymous sites. The protein sequences encoded by the first three exons of the variant *cd4.1* alleles are shown in (**B**). Polymorphic amino acids are shaded, and a consensus sequence logo is shown underneath the alignment. The location of the signal peptide and Ig-like fold domain predicted by InterProScan is indicated above the alignment; shaded bars indicate the exons encoding the corresponding amino acids

Recognition of MHC2-peptide complexes by T cells is facilitated by a CD4 co-receptor expressed by helper and regulatory T cells [34]. Most teleost fish species have two CD4 genes, *cd4.1* and *cd4.2* [35], and we searched for evidence of these genes in *O. cubifrons*. Expression of both *cd4.1* and *cd4.2* orthologues could be readily detected in *O. cubifrons* RNA-seq data, and sequences of the complete coding regions of these orthologues was determined (Table 1). Examination of the inferred protein sequence of the intracellular tails of CD4.1 and CD4.2 revealed the presence of the expected CxC motif, which is required for interaction with Lck, in both instances (Fig. 5B C). A membrane-proximal arginine/lysine rich region, thought to facilitate interactions with Lck [36, 37], was also observed in the intracellular tails of CD4.1 and CD4.2 (Fig. 5B C). Analysis of the 5’ coding region of the *O. cubifrons cd4.1* transcripts (encoding the CD4.1 N-terminus) however revealed an unexpectedly high degree of sequence variability, primarily within the second and third coding exons. This variation was observed in both specimens for which sequence data was available, and through the use of a combination of RNA-seq data, whole-genome shotgun sequences, and targeted Sanger sequencing we identified three *cd4.1* variants (V1 – V3), based upon the sequence of the first three coding exons (Fig. 6). V1 was unique to the first specimen, V2 was shared by both, and V3 was unique to the second specimen. Alignment of these three variants revealed a total of 17 polymorphic sites within the first three coding exons; 16 SNPs and one 3 bp indel (Fig. 6A). Among the SNPs, 14/16 were at non-synonymous sites, and all polymorphic sites were found within a region predicted by InterProScan [38] to encode an immunoglobulin-like fold (Fig. 6B). All of the variants are expected to encode full-length proteins (no non-sense mutations were detected, and the 3 bp indel preserved the translational reading frame), however the functional consequence of the sequence variation within the N-terminal Ig-domain are unknown. Despite this uncertainty, the expression of genes involved in antigen presentation (*mhc2a*, *mhc2b* and *cd74*), together with the detection of *cd4.1* and *cd4.2* co-receptor genes, indicates that the MHC2-pathway is active in *O. cubifrons*.

## Discussion

Our analysis of immune genes demonstrates that the batfish *O. cubifrons* possesses the core genes required to mount adaptive T and B cell responses. From an immunological perspective, compared to other members of the *Lophiiformes*, *O. cubifrons* therefore has a similar gene complement to representatives from *Antennaroidei* and *Chaunacoidei*, but differs from the *Lophioidei* and *Ceratioidei*. As mentioned above, within the Lophiiformes, the loss of adaptive immune genes correlates with reproductive mode, and with the exception of the *Lophioidei*, only species that utilise temporary or permanent attachment-based mating strategies have so far exhibited reductions in their adaptive immune gene complement. The spawning and courtship behaviour of *O. cubifrons* has been observed in captivity, and while males were observed to nip spawning females during courtship, no pair attachment was documented [39], and from this perspective, the full complement of core adaptive immune genes is consistent with their “non-attaching” phenotype.

One curious observation from the analysis of *O. cubifrons* adaptive immune genes is the high degree of polymorphism observed in the *cd4.1* gene - interestingly, this variability was focused within the sequence encoding the N-terminal Ig-domain, which in mammals is known to be the domain that interacts directly with MHC2 [40, 41]. Variation in this domain has previously been noted in several primate species, where it is thought to provide protection against lentiviruses that use CD4 as a receptor to gain entry into the cell [42–44]. To date we have only had the opportunity to analyse *cd4.1* sequences from two *O. cubifrons* individuals, so it is not yet possible to make firm conclusions regarding the level of *cd4* diversity at the population level in this species. Nevertheless, the detection of three different alleles in two individuals, the clustering of the polymorphic sites in a single structural domain, and the high proportion of non-synonymous SNPs, is indicative of a selective process worthy of further investigation, especially in light of the complete loss of CD4 observed in some closely related species.

Within the *Lophiiformes*, complete loss of *cd4* genes has been noted in *L. piscatorius*, as well as in four species in the Ceratioid sub-order (*G. vanhoeffeni*, *C. couesii*, *H. mollis*, and *P. spiniceps*), and fails to correlate solely with reproductive mode (with examples of loss in non-attachers, temporary attachers and permanent multi-attachers). Furthermore, loss of *cd4* genes is not unique to members of the *Lophiiformes*, and was first reported in the Atlantic cod [45], as well as in several species of pipefish [46]. At present no single unifying feature appears sufficient to explain the absence of CD4 in these fish species, and this remains an important open question that requires clarification [47]. It is tempting to speculate that CD4 variability in *O. cubifrons* may be evidence for an active selective process driving CD4 evolution in this species. However, testing this proposal will require sequence data from a greater number of individuals, ideally paired with data from other closely related batfish species.

## Conclusions

To the best of our knowledge, this is the first survey of a batfish immunogenome, and the results demonstrate that this fish species has the canonical complement of adaptive immune genes. The *Ogcocephaloidei* sub-order is complex, containing at least 9 genera and over 60 species [48, 49], and a limitation of our study is that we have so far only been able to analyse a single representative species of the sub-order; further sampling is therefore needed to determine if the immunogenome of *O. cubifrons* is representative for the entire sub-order, or if other immune configurations are present. Despite this limitation, the data presented here give the first insight into adaptive immunity in this sub-order, and the sequence data generated in this study will be a useful resource for research in both immunological and non-immunology-related fields.

## Methods

### Specimens and sequencing

Two *Ogcocephalus cubifrons* specimens, designated Ocub1 (male) and Ocub2 (unknown sex), were obtained from an ornamental fish supplier, and euthanised by immersion in a 3 L bath containing a lethal dose of MS-222 (Tricaine, 100 mg/L, diluted in artificial seawater), in accordance with local animal welfare regulations. Thymus and testes were harvested immediately for RNA- and genomic DNA-extraction respectively. RNA was extracted from thymus tissue using the TRI Reagent (Merck/Sigma-Aldrich) method, as per the manufacturer’s instructions. RNA-sequencing libraries were prepared using the Illumina TruSeq Stranded mRNA Library Prep Kit, and then sequenced on an Illumina NovaSeq 6000 (Ocub1) or NextSeq 500 (Ocub2) sequencer. Genomic DNA was isolated from testis tissue by digestion with proteinase K, and subsequent phenol-chloroform extraction. Genomic sequencing libraries were prepared using the Illumina TruSeq PCR-Free Library Prep Kit, and sequenced on an Illumina NovaSeq 6000 sequencer. RNA was sequenced from both Ocub1 and Ocub2 thymi; genomic sequencing was performed on Ocub1 only. All sequencing data has been deposited into the NCBI sequence read archive (SRA) and can be accessed under the BioProject ID PRJNA905091. To validate the species identity of the sequenced individuals, a complete Cytochrome c oxidase I (COI) gene sequence was assembled from both Ocub1 and Ocub2 sequence data, and used to search the BOLD Identification System species level barcode records [50]. The top three hits in both instances were to previously existing *O. cubifrons* barcodes, which exhibited 100–99.85% identity to the search sequences. To estimate genomic coverage we performed SPAdes[51] *de novo* assembly, followed by BUSCO[52] (version 5.4.2) assessment, which yielded the following output: C:47.5%[S:46.0%,D:1.5%],F:14.5%,M:38.0%,n:3640 (where C = complete, S = complete and single-copy, D = complete and duplicated, F = fragmented, M = missing and n = total BUSCO groups searched; the actinopterygii_odb10 lineage dataset was used). A similar approach was used to estimate transciptome coverage. Transcriptomes were assembled *de novo* using Trinity [53] (version 2.8.5), and the pooled thymus transciptome (Ocub1 and Ocub2) was subjected to BUSCO assessment, which yielded the following output: C:85.5%[S:25.0%,D:60.5%],F:2.9%,M:11.6%,n:3640. The *de novo* assemblies are available from the authors upon request.

In some instances, specific targets were validated by Sanger sequencing; in these cases, target amplicons were amplified using Q5 High-Fidelity DNA Polymerase (New England Biolabs) and the primers listed in Table 2. Amplicons were subsequently cloned into pJET1.2 using the CloneJET PCR Cloning Kit (ThermoFischer Scientific), and clones of interest were sequenced using the ABI PRISM BigDye Terminator v3.1 Ready Reaction Cycle Sequencing Kit, and reactions were analysed on an Applied Biosystems 3730xl DNA Analyzer.

**Table 2:** PCR primers used in this study

Target	Primer 1	Primer 2	Use
*cd4.1*, exons 1–3	TCTTTGGTGTGCTTTGTGCC	TGTGCGTCCTCTTACCACTG	amplification and cloning of *cd4.1* variant region
*mhc1*	GAGGAGGGCGGATAAACGAG	TCAGGGTGGTGTGTTTGTCG	amplification and cloning of *mhc1* variants
*mhc2b*	CATGGCTTCATCCTGTCTCACC	GATCAGGTCCAGGACTGCTC	amplification and cloning of *mhc2* variants
*rag1*	GTTCGCAGACGAGAGGGAG	GAGGAACGTGTTGACCCGAA	verification of genomic assembly
*rag1* – *rag2* interval	TGATGGATGGTTCCTCGCTC	TTTAGGGAGGGTCCGTGTGA	verification of genomic assembly

### Sequence analysis and identification of orthologues

Comparisons were made primarily with sequences derived from *T. rubripes*, as this species belongs to the *Tetraodontiformes*, an order closely related to the *Lophiiformes* [54], and are known to possess the adaptive immune genes examined in this study [55–60]. *T. rubripes* protein sequences were used to search *O. cubifrons* transcriptome and genome databases, using a SequenceServer 2.0.0 [61] custom BLAST server, and hits were then assembled using DNASTAR SeqMan Ultra software. The resulting *O. cubifrons* transcripts, and their inferred protein sequences, were then BLASTed against both *T. rubpripes*- and teleost-specific databases (NCBI taxids 31,033 and 32,443 respectively) to confirm the identity of the candidate orthologue [62, 63]. DNA and protein alignments were performed with DNASTAR MegAlign Pro software (version 17.2.1), using the MUSCLE [64] or ClustalW [65] algorithms. Transmembrane regions in proteins were predicted with TMHMM 2.0 [66], and signal peptides and domain predictions were made using InterProScan [38]. Phylogenetic trees were constructed in MegAlign Pro using the RAxML method [67].

## Electronic supplementary material

Below is the link to the electronic supplementary material.

Additional File 1: Supplementary Figure 2. Phylogenetic analysis of putative *O. cubifrons* Dntt and Polymerase μ (Polm) orthologues.

Additional File 2: Supplementary Figure 3. Assessment of *O. cubifrons* AID regions predicted to modulate catalytic function.

Additional File 3: Supplementary Figure 4. MHC1 sequence diversity.

Additional File 4: Supplementary Figure 5. MHC2b sequence diversity.

Additional File 5: Supplementary Figure 1. Overview of adaptive immune system gene loss in anglerfish suborders.

## Data Availability

The datasets generated and/or analysed during the current study are available in the NCBI Sequence Read Archive (SRA) repository under the BioProject ID PRJNA905091. Assembled sequences generated in this study are available in the NCBI GenBank repository, or from the corresponding author on reasonable request.

## Declarations

**Ethics approval and consent to participate**.

This animal study was approved by the Animal Welfare Committee of the Max Planck Institute for Immunobiology and Epigenetics. All animal experiments were conducted in accordance with German state and federal guidelines and regulations. The methods are reported in accordance with ARRIVE guidelines.

**Consent for publication**.

### Corresponding author

Correspondence to Jeremy Swann (swann@ie-freiburg.mpg.de).
