# Supplementary material for: A survey of the adaptive immune genes of the polka-dot batfish Ogcocephalus cubifrons
Source: BMC Immunol. 2023 Jul 21;24:20. doi: 10.1186/s12865-023-00557-0 (PMC10362645; doi:10.1186/s12865-023-00557-0)
Supplement: Supplementary file 1 — Additional File 1: Supplementary Figure 2. Phylogenetic analysis of putative O. cubifrons Dntt and Polymerase μ (Polm) orthologues. [file 12865_2023_557_MOESM1_ESM.pdf]

Supplementary Figure 2

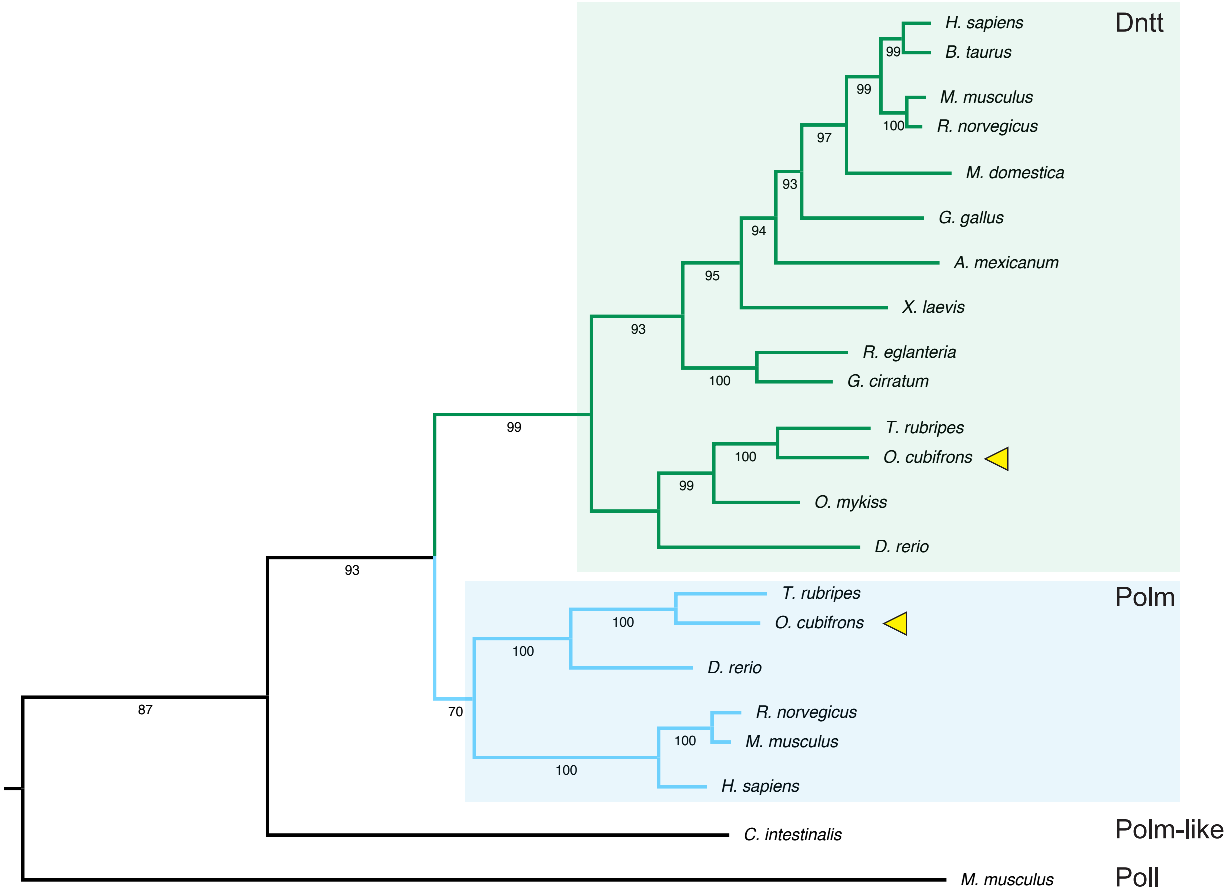

Supplementary Figure 2. *Phylogenetic analysis of putative O. cubifrons Dntt and Polymerase μ (Polm) orthologues.* The inferred protein sequences of candidate *O. cubifrons* Dntt and Polm proteins were aligned with the set of validated Dntt and Polm proteins reported in [15], using the ClustalW algorithm, and a phylogenetic tree was constructed using the RAxML method, performed in MegAlign Pro. Mouse polymerase λ (Poll) was used to root the tree. Numbers indicate bootstrap percentages after 1000 iterations. Note that Dntt and Polm sequences segregate into two distinct branches, indicated in green and blue respectively. Species names are given at the tip of each branch; yellow arrowheads highlight the location of the proposed *O. cubifrons* Dntt and Polm orthologues within the tree.
