# Supplementary material for: A survey of the adaptive immune genes of the polka-dot batfish Ogcocephalus cubifrons
Source: BMC Immunol. 2023 Jul 21;24:20. doi: 10.1186/s12865-023-00557-0 (PMC10362645; doi:10.1186/s12865-023-00557-0)
Supplement: Supplementary file 2 — Additional File 2: Supplementary Figure 3. Assessment of O. cubifrons AID regions predicted to modulate catalytic function. [file 12865_2023_557_MOESM2_ESM.pdf]

Supplementary Figure 3

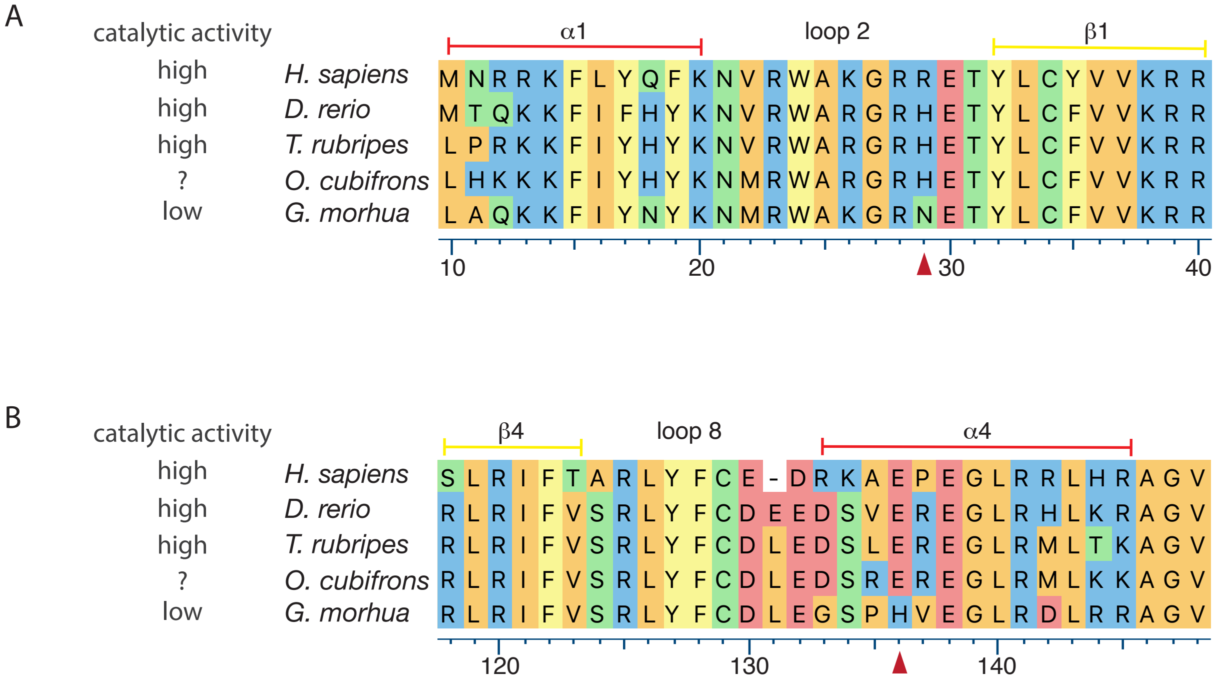

Supplementary Figure 3. *Assessment of O. cubifrons AID regions predicted to modulate catalytic function.* Sub-regions of the inferred *O. cubifrons* AID protein were aligned with the corresponding regions of three AID proteins with high catalytic activity (human, zebrafish and pufferfish) and one AID protein with low catalytic activity (cod), as defined in [16]. The low activity of cod AID is partially due to the presence of an asparagine residue at position 29 (panel A) and a histidine residue at position 136 (panel B). In contrast, the *O. cubifrons* AID protein encodes amino-acids associated with high activity at the respective positions (29H and 136E). Alpha-helix, loop, and beta-strand regions of the proteins are indicated above the alignments, and red arrowheads below the alignment mark the location of the key residues that modulate catalytic activity.
