# Supplementary material for: A survey of the adaptive immune genes of the polka-dot batfish Ogcocephalus cubifrons
Source: BMC Immunol. 2023 Jul 21;24:20. doi: 10.1186/s12865-023-00557-0 (PMC10362645; doi:10.1186/s12865-023-00557-0)
Supplement: Supplementary file 3 — Additional File 3: Supplementary Figure 4. MHC1 sequence diversity. [file 12865_2023_557_MOESM3_ESM.pdf]

Supplementary Figure 4

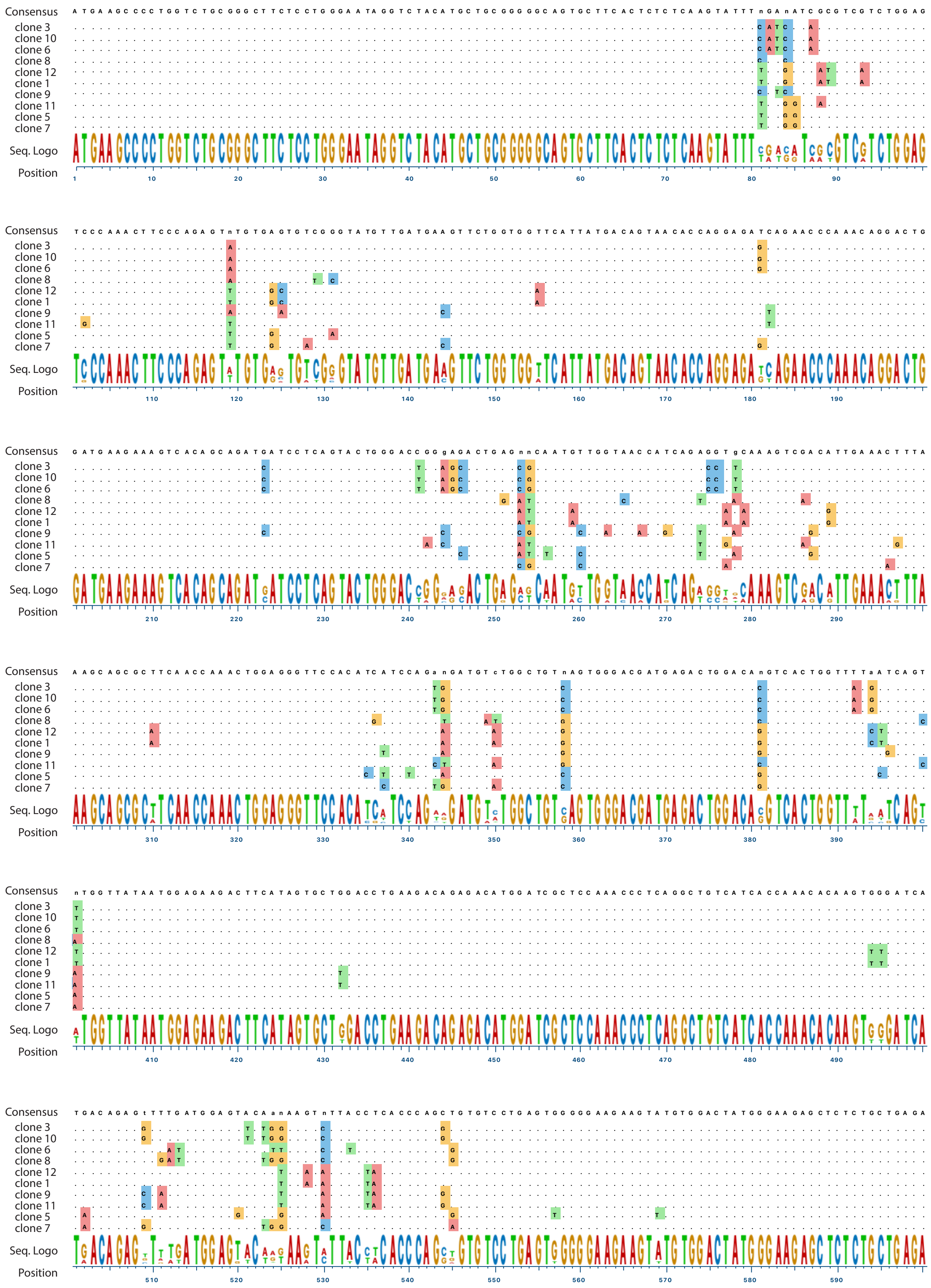

Supplementary Figure 4 (continued)

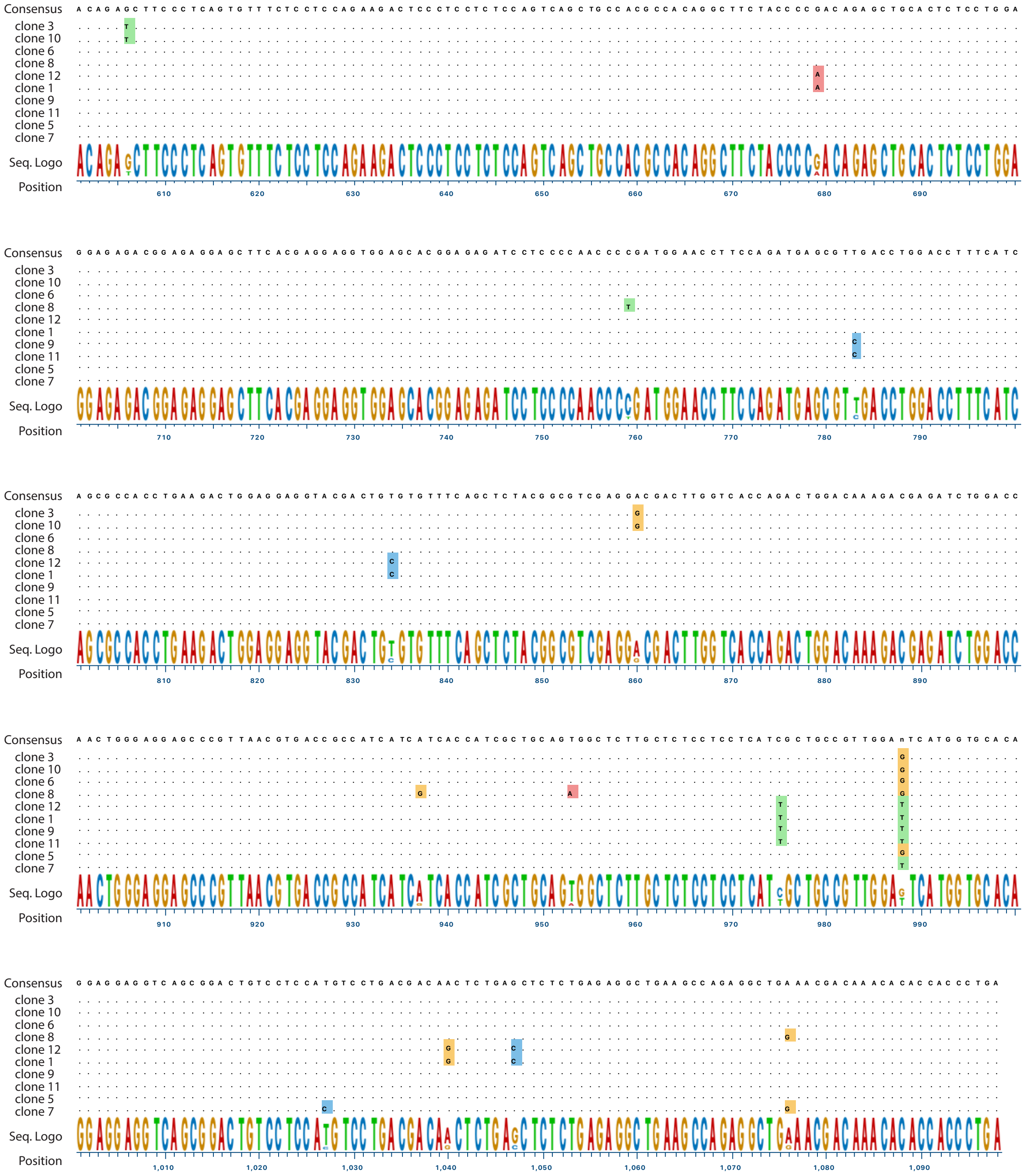

Supplementary Figure 4. *MHC1 sequence diversity.*
An alignment of the complete coding sequences of 10 Sanger-sequenced mhc1 clones is depicted. A consensus sequence is shown above the alignment; in the aligned sequences a dot indicates identity with the consensus, bases that differ from the consensus are shown, and shaded to highlight differences. Clones 3 & 10 are identical, as are 1 & 12. In clone 5 a C>T mutation at position 340 generates an in-frame stop codon, indicating that this is likely to be a non-functional variant. Note also that at several positions in the alignment three different bases are present, indicating that a minimum of at least three distinct alleles must be present overall.
