# Supplementary material for: A survey of the adaptive immune genes of the polka-dot batfish Ogcocephalus cubifrons
Source: BMC Immunol. 2023 Jul 21;24:20. doi: 10.1186/s12865-023-00557-0 (PMC10362645; doi:10.1186/s12865-023-00557-0)
Supplement: Supplementary file 4 — Additional File 4: Supplementary Figure 5. MHC2b sequence diversity. [file 12865_2023_557_MOESM4_ESM.pdf]

Supplementary Figure 5

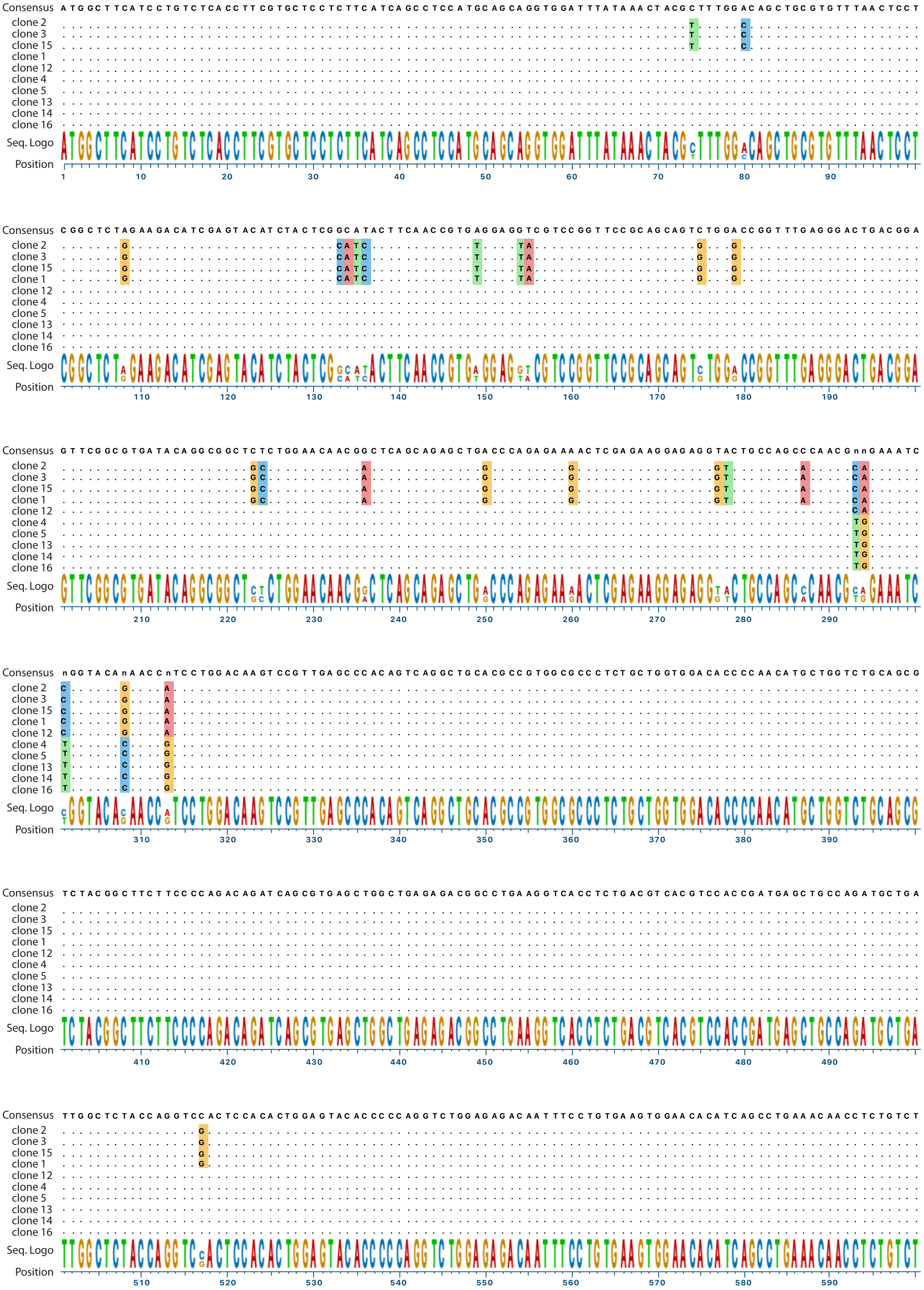

Supplementary Figure 5 (continued)

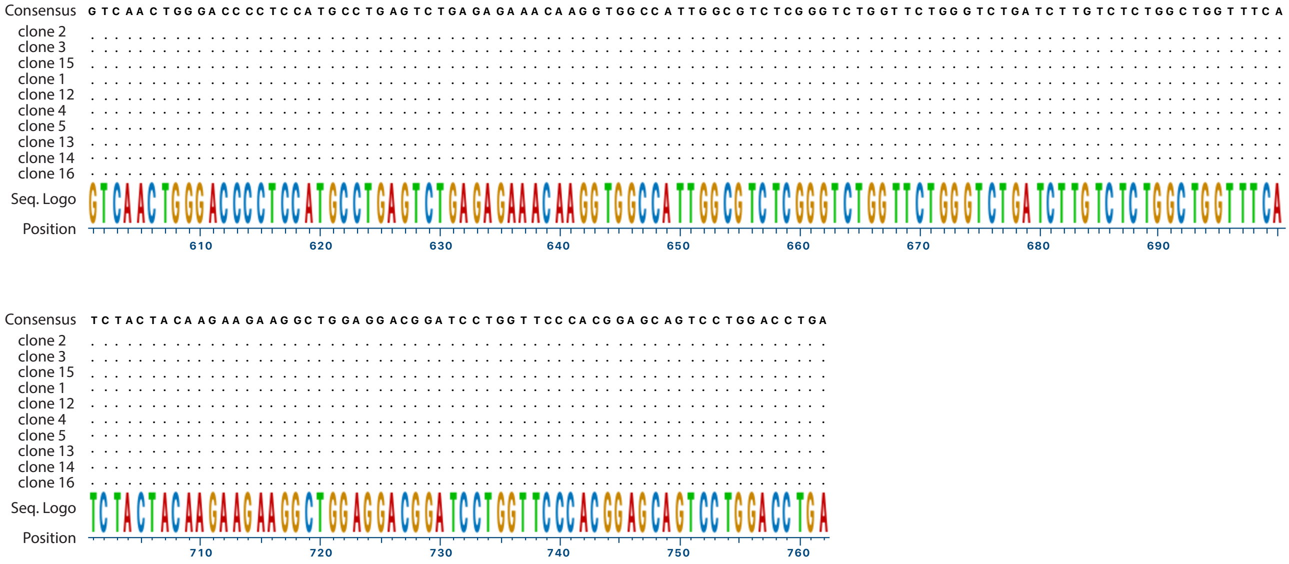

Supplementary Figure 5. *MHC2b sequence diversity.*
An alignment of the complete coding sequences of 10 Sanger-sequenced mhc2b clones is depicted. A consensus sequence is shown above the alignment; in the aligned sequences a dot indicates identity with the consensus, bases that differ from the consensus are shown, and shaded to highlight differences. Clones 2, 3 and 15 are identical, as are clones 4, 5, 13, 14 and 16. Clones 1 and 12 are unique, but are potentially PCR-induced chimeras, as they share features with both of the other sets of clones, and have no distinct sequence characteristics that definitively distinguish them from the other clone groups. Note that a maximum of two different bases are present at any single site in the alignment.
