# Supplementary material for: A survey of the adaptive immune genes of the polka-dot batfish Ogcocephalus cubifrons
Source: BMC Immunol. 2023 Jul 21;24:20. doi: 10.1186/s12865-023-00557-0 (PMC10362645; doi:10.1186/s12865-023-00557-0)
Supplement: Supplementary file 5 — Additional File 5: Supplementary Figure 1. Overview of adaptive immune system gene loss in anglerfish suborders. [file 12865_2023_557_MOESM5_ESM.pdf]

Supplementary Figure 1

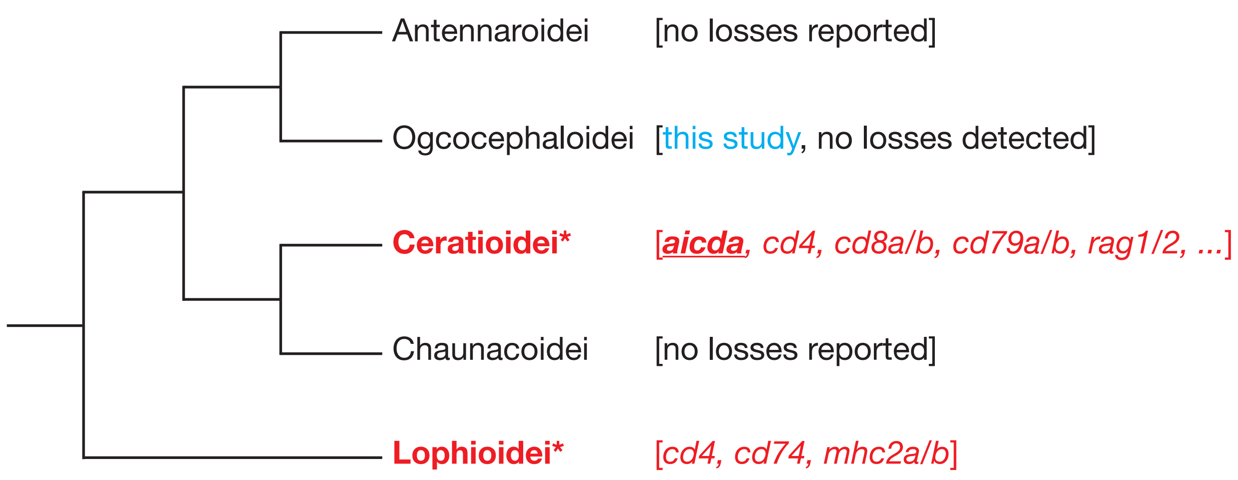

Supplementary Figure 1. *Overview of adaptive immune system gene loss in anglerfish suborders.* A stylised phylogenetic tree representing the subordinal relationships of the five anglerfish suborders is shown (adapted from [13]). Suborders in which the loss of adaptive immune genes has been reported are highlighted in red and marked with an asterisk (*). Examples of missing adaptive immune genes are listed on the right. Note that in the case of the ceratioid suborder only *aicda* (bold, underlined) is missing from all representative species examined to date; the other example genes are lost only in particular species or genera, see [4] for details.
